# Development and Evaluation of Nanoparticles-in-Film Technology to Achieve Extended In Vivo Exposure of MK-2048 for HIV Prevention

**DOI:** 10.3390/polym14061196

**Published:** 2022-03-16

**Authors:** Xin Tong, Sravan Kumar Patel, Jing Li, Dorothy Patton, Elaine Xu, Peter L. Anderson, Urvi Parikh, Yvonne Sweeney, Julie Strizki, Sharon L. Hillier, Lisa C. Rohan

**Affiliations:** 1 Department of Pharmaceutical Sciences, School of Pharmacy, University of Pittsburgh, Pittsburgh, PA 15213, USA; x.o.tong@pitt.edu (X.T.); patels10@mwri.magee.edu (S.K.P.); jil132@pitt.edu (J.L.); elx2@pitt.edu (E.X.); 2 Magee-Womens Research Institute, Pittsburgh, PA 15213, USA; ump3@pitt.edu (U.P.); hillsl@mwri.magee.edu (S.L.H.); 3 Department of Obstetrics and Gynecology, University of Washington, Seattle, WA 98195, USA; dpatton@uw.edu (D.P.); ytcs@uw.edu (Y.S.); 4 Department of Pharmaceutical Sciences, Skaggs School of Pharmacy and Pharmaceutical Sciences, University of Colorado, Aurora, CO 80045, USA; peter.anderson@cuanschutz.edu; 5 Merck & Co., Inc., 2000 Galloping Hill Road, Kenilworth, NJ 07033, USA; julie.strizki@merck.com; 6 Department of Obstetrics, Gynecology, and Reproductive Sciences, School of Medicine, University of Pittsburgh, Pittsburgh, PA 15213, USA

**Keywords:** nanomedicine, vaginal drug delivery, HIV/AIDS, antiretroviral, nonhuman primates

## Abstract

MK-2048 is a second-generation integrase inhibitor active against HIV, which has been applied vaginally using ring formulations. In this work, a nanoparticle-in-film technology was developed as a discrete pre-exposure prophylactic product option against HIV for an extended duration of use. A film platform loaded with poly (lactic-co-glycolic acid) nanoparticles (PNP) encapsulating MK-2048 was engineered. MK-2048 PNPs were loaded into films that were manufactured via the solvent casting method. Physicochemical and mechanical properties, in vitro efficacy, *Lactobacillus* compatibility, in vitro and ex vivo permeability, and in vivo pharmacokinetics in macaques were evaluated. PNPs with a mean diameter of 382.2 nm and −15.2 mV zeta potential were obtained with 95.2% drug encapsulation efficiency. PNP films showed comparable in vitro efficacy to free MK-2048 (IC_50_ 0.46 vs. 0.54 nM) and were found to have no impact on *Lactobacillus*. MK-2048 encapsulated in PNPs showed an increase in permeability (>4-fold) compared to the free MK-2048 in MDCKII cell lines. Furthermore, PNPs had higher ectocervical tissue permeability (1.7-fold) compared to free MK-2048. PNP films showed sustained drug levels for at least 3 weeks in the macaque vaginal fluid. This work demonstrates the synergy of integrating nanomedicine and polymeric film technology to achieve sustained vaginal drug delivery.

## 1. Introduction

Despite global HIV testing and treatment programs, there were an estimated 37.7 million new infections recorded in 2020 [1]. Of these, about 53% of infections were in women, including adolescents and young girls (aged 15–24 years), showing the disproportionate impact in women due to social, cultural, economic, and biological vulnerabilities [2]. The major mode of new HIV-1 infections in women worldwide is through sexual transmission [3].

Currently, there are two orally administrated antiretroviral (ARV) drug combinations on the market, Truvada and Descovy, for pre-exposure prophylaxis (PrEP). However, Descovy has not been approved for use by cisgender women although clinical trials in women are underway. Uptake and persistence with daily pill-taking by women have been low due to a range of barriers [4,5,6]. Adherence to product use plays a critical role in maintaining drug exposure required for prevention effectiveness [7,8]. Therefore, to better protect women from HIV infection, it is critical to design products from a woman’s perspective [7,8]. To date, several product types have been investigated within this target population, namely, vaginal rings [9,10], tablets [11], hydrogels [12,13], and polymeric films [14,15]. Polymeric films have advantages, such as discreet use, ease of self-administration, low cost, and non-leakiness, which makes them one of the attractive and viable vaginal drug delivery systems [16,17]. In a study that investigated the preferred physical characteristics for the design of vaginal films for HIV prevention [18], women identified that it was optimal for films to be smooth, thin, and translucent. Thus, within this work target film product characteristics included flexibility, softness, and no sharp edges to enhance acceptability and product insertion [17].

MK-2048 is a second-generation integrase strand transfer inhibitor (INSTI) that has been evaluated in two Phase 1 studies in a vaginal ring formulation [19,20]. It has potent activity against wild-type and raltegravir-resistant HIV variants [19,20,21]. In a clinical study that evaluated MK-2048 vaginal rings (MTN-027), it was found that tissue-associated drugs did not correlate with HIV inhibitory activity in an ex vivo challenge model [19]. Reasons for this result could be insufficient drug penetration or possible drug efflux out of the tissue. Literature had shown other integrase inhibitors such as raltegravir and elvitegravir are substrates of efflux transporters [22,23,24,25], including P-glycoprotein (P-gp), and Breast Cancer Resistance Protein (BCRP). Meanwhile, our group has preliminary data indicating that MK-2048 is a substrate for efflux transporters existing in the cervicovaginal tissue. This suggests that a strategy that includes evading these efflux transporters may be optimal. Even though MK-2048 may not have optimal characteristics for vaginal delivery because it is effluxed, this molecule was incorporated in the present study as a model ARV drug for the evaluation of a nanoparticle-based film formulation.

Our goal in this work was to develop an alternate vaginal dosage form with an extended duration of protection from HIV infection while providing a discreet product option for women. Nanoparticles have tuneable properties for drug release and can enhance tissue access by overcoming drug efflux transporters [26]. Nanoparticles, combined with other drug delivery systems, can also achieve extended-release [27]. Previous work demonstrated that the nanoparticle-in-film technology can achieve extended-release of anti-HIV drugs such as tenofovir for 24 h in an in vivo mouse model [28,29]. In order to reduce dosing frequency and potentially reduce adherence and acceptability issues observed in HIV prevention trials [30,31], there is a need to further extend the drug release. Therefore, a nanoparticle-incorporated vaginal film platform was designed to achieve extended-release (ER) for MK-2048 and was evaluated in in vitro, ex vivo, and in in vivo nonhuman primate models. 

## 2. Materials and Methods

### 2.1. Materials

MK-2048 (purity > 99%) was provided by Merck & Co., Inc. (Kenilworth, NJ, USA). Poly (lactic-co-glycolic acid) (PLGA) with molar ratios of 50:50 lactic acid: glycolic acid was purchased from Sigma-Aldrich (Resomer 503 [Mw 24,000–38,000 Da]; St. Louis, MO, USA). Chemical reagents for nanoparticle preparation and analytical assays were purchased from Fisher Scientific (Pittsburgh, PA, USA). Polyethylene glycol (PEG) 8000 was purchased from Spectrum (Gardena, CA, USA). Hydroxypropyl methylcellulose (HPMC; METHOCELTM E5) was obtained from Dow Chemical Company (Midland, MI, USA). Polyvinyl alcohol 4-88 (PVA) was purchased from Millipore Sigma (Temecula, CA, USA). Cell culture reagents were obtained from GIBCO, Invitrogen by Life Sciences, Inc. (Lenexa, KS, USA). Ultrapure water was prepared by passing distilled water through a Milli-Q Reagent Water System (Millipore, Burlington, MA, USA). All the other chemical reagents for film preparation were purchased from Fisher Scientific (Pittsburgh, PA, USA). 

### 2.2. Production and Characterization of the MK-2048-Loaded PLGA Nanoparticles (PNP)

An emulsion evaporation method was used to prepare MK-2048-loaded PLGA Nanoparticles (PNP). Briefly, 1.92 g of PVA was dissolved in 96 mL of Milli-Q water to form the 2% PVA solution (aqueous phase). Then, the MK-2048 (2.4 g) and PLGA (4.8 g) were dissolved in 48 mL methylene chloride successively, as the oil phase. The oil phase was then added into the aqueous phase under sonication (Vibra-Cell Sonics with S6 probe, Sonics & Materials, Inc., Newtown, CT, USA) for 13 cycles (10 s on/2 s off for each cycle; 30 s gap between cycles) with 30% amplitude. The oil-in-water emulsion was later diluted with Milli-Q water and kept on an ice bath and under magnetic stirring overnight at 200 rpm. PNPs were concentrated by centrifugation (15,000× *g* at 4 °C for 15 min). Excessive PVA was washed off twice with Milli-Q water. PNPs were finally reconstituted with 5% trehalose solution and frozen overnight at −80 °C before lyophilization. The lyophilized PNPs were characterized for hydrodynamic diameter (dispersed in water), poly-dispersion index (PDI), and zeta-potential using a ZetaSizer Nano ZS (Malvern Panalytical Ltd., Malvern, UK). The size of PNPs was reported as the average value from three measurements. The appearance of PNPs was evaluated by a JEOL JEM-2100F (JEOL Ltd., Tokyo, Japan) transmission electron microscopy (TEM) and a Zeiss SIGMA VP (Carl Zeiss AG, Jena, Germany) scanning electron microscopy (SEM). 

The percentage of drug encapsulation efficiency (EE%) was estimated (Equation (1)) by assaying the amount of MK-2048 (active pharmaceutical ingredient, API) in supernatants recovered during purification by reverse-phase high-performance liquid chromatography (RP-HPLC) with UV detection.

(1) $$\mathrm{Encapsulation}\;\mathrm{Efficiency}\;\left(\mathrm{EE}\%\right)=1-\frac{\mathrm{API}\;\mathrm{tested}\;\mathrm{in}\;\mathrm{the}\;\mathrm{supernatant}}{\mathrm{Total}\;\mathrm{API}\;\mathrm{used}}\times 100\%$$

### 2.3. High-Performance Liquid Chromatography (HPLC) Method for the Analysis of MK-2048

Dionex Ultimate 3000 HPLC system (Thermo Fisher, Waltham, MA, USA) and Chromeleon data acquisition system was used. The column used was the Xbridge C18 HPLC column (5 µm, 2.1 × 50 mm, Waters, Milford, MA, USA) with Guard Column (Phenomenex, Torrance, CA, USA). The mobile phases consist of (A) aqueous phase: 0.1% of formic acid in Milli-Q water, and (B) organic phase: acetonitrile. Gradient elution with a 1 mL/min flow rate was used for separation. The gradient is as follows: at 0–2 min, 10% B; 2–6 min, a linear change to 50% B; 6–7 min, 50% B; 7–8 min, a linear change to 10% B; 8–11 min, 10% B. 10 µL of the sample was injected in duplicate. MK-2048 can be detected at a wavelength of 334 nm. Drug Loading (DL%) was calculated using the ratio of MK-2048 detected in PNPs/PNP films to the measured PNP/ PNP film weights, respectively. 

### 2.4. Liquid Chromatography-Mass Spectrometry (LC/LC-MS) Method for the Bioanalysis of MK-2048

For the determination of MK-2048 in the in vivo study, Acquity UPLC (Waters Corporation, Milford, MA, USA) and ThermoFisher Scientific Ultra and Vantage MS/MS (Waltham, MA, USA) instruments were utilized for LC/LC-MS, with C18 columns (Waters Acquity C18 BEH UPLC column, 1.7 μm, 2.1 mm× 100 mm). The XCalibur 2.2 software was used to control the LC-MS/MS equipment and to analyze the data. The mobile phases were 90% Acetonitrile: 10% 10 mM Ammonium Formate pH 3 (mobile phase A) and 40% Acetonitrile: 60% 10 mM Ammonium Formate pH 3 (mobile phase B). The flow rate was set to 0.5 mL/min. Each injection took three minutes to complete. MK-2048 (m/z 462.3) and MK-2048 internal standard (m/z 468.3) were detected in the positive ion mode (ESI+) with a spray voltage of 3000 V and a capillary temperature of less than 250 °C. The calibration range was 25 pg/mL to 50,000 pg/mL.

For the determination of MK-2048 in the in vitro and ex vivo studies, Acquity UPLC and Xevo TQ-S MS/MS instruments (Waters Corporation, Milford, MA, USA) were utilized for LC/LC-MS, with C18 columns (Waters Acquity C18 BEH UPLC column, 1.7 μm, 2.3 × 50 mm). The MassLynx 4.1 software was used to control the LC-MS/MS equipment and to analyze the data. The mobile phases were 0.1% formic acid in Milli-Q water (mobile phase A, 60%) and 0.1% formic acid in acetonitrile (mobile phase B, 40%). The flow rate was set to 0.4 mL/min. Each injection took four minutes to complete. MK-2048 (m/z 462,143) was detected in the positive ion mode (ESI+) with a spray voltage of 3000 V and a capillary temperature of less than 250 °C. Collected samples were diluted 1:1 in Milli-Q water with 50% acetonitrile and injected at a volume of 5 µL.

### 2.5. Preparation and Characterization of PNP-Incorporated Polymeric Films

PNP-incorporated polymeric films (PNP films) were manufactured using the solvent-cast method. A fast-dissolving film formulation was used as the base formulation for PNP film preparation. Briefly, polyvinyl alcohol (PVA) was dissolved in Milli-Q water at 90 °C under an overhead mixer, followed by adding HPMC and PEG 8000 subsequently until a homogenous blend was formed. Plasticizers (propylene glycol and glycerin) were added to this blend at a low speed (50 rpm). Lyophilized PNP powder, which was processed mildly in a mortar and pestle to break lumps, was added into the polymer solution. The prepared blend was coated onto a substrate fitted to a Thin Film Applicator (Elcometer, Manchester, UK) at a thickness of 110 µm. The film sheet dried at 71 °C for 15–20 min was peeled off from the substrate, cut into 1” × 1” units, and packed in pouches for further characterization.

Film appearance was tested by visual observations. Shape, color, and surface texture were recorded. Individual film weight and thickness were measured using an analytical balance (Mettler Toledo XS105, Columbus, OH, USA) and a thickness gauge (Mitutoyo Corporation, Kanagawa, Japan), respectively. Puncture Strength (PS) was characterized with a texture analyzer (TA.XT.Plus, Hamilton, MA, USA). Water Content (WC) was measured using Karl-Fisher apparatus (Metrohm, 758KFD Titrino, Herisau, Switzerland) in accordance with the titration method specified by the manufacturer. Drug content was determined by RP-HPLC (described in Section 2.3) after overnight extractions with 40% acetonitrile in water. 

### 2.6. PNP Film Compatibility with Lactobacillus

The standard vaginal microbicide safety test was performed as previously described [32] to assess *Lactobacillus* compatibility with MK-2048 PNP film. Briefly, bacteria were grown for 24 h in 6% CO_2_, 37 °C, on Columbia agar 5% sheep blood plates (BA). Film samples were prepared by dissolving MK-2048 PNP film in 5 mL of N-2-aminoethanesulfonic acid (ACES) buffer. Four strains of *Lactobacillus,* namely *L. crispatus* CTV05, *L. crispatus* ATCC 33197, *L. jensenii* ATCC 25258, and *L. jensenii* LBP 28Ab, were exposed to either ACES buffer alone or PNP film solution for 30 min. The mixture was serially diluted and plated on BA for colony counts. After 2 days, in 6% CO_2_, 37 °C, viability was determined by calculating the log value with standard plate counts. For each bacteria strain, log value differences were calculated between the film dissolution group and the control group (ACES buffer only). A sample passes the compatibility test if the log difference was less than (<) 1.0.

### 2.7. Cell Culture

The transporter-overexpressing cell lines (MDCKII BCRP and MDR1) were kindly provided by Dr. Philip Empey from the University of Pittsburgh School of Pharmacy, and the TZM-bl cell line was purchased from American Type Culture Collection (ATCC, Manassas, VA, USA). The MDCKII BCRP and MDR1 cells were cultured in Dulbecco’s Modified Eagle Medium (DMEM) with the addition of 10% bovine fetal serum (FBS), 100 U/mL penicillin, and 100 μg/mL streptomycin at 37 °C in 5% of carbon dioxide (CO_2_). The TZM-bl cells were cultured in DMEM with 10% FBS, 100 U/mL penicillin, 100 μg/mL streptomycin, and 1% 200 mM l-glutamine at 37 °C in 5% CO_2._

### 2.8. PNP Film Efficacy Study against HIV-1_BaL_ in the TMZ-bl Antiviral Assay

Cytotoxicity of MK-2048-loaded PNP film was evaluated in TZM-bl cells. Cells were seeded in a 96-well plate at 1 × 10^4^ cells/well overnight. Films were dissolved in 1 mL saline solution and serial dilutions of film solution were incubated with TZM-bl cells at 37 °C in 5% CO_2_. After 48 h luminescence was measured using CellTiter-Glo (Promega, Madison, WI, USA). The % viability of cells treated with film solution was calculated by comparing luminescence of film-treated cells to that of cells without any treatment.

The in vitro efficacy studies were conducted as previously described [33]. TZM-bl cells were plated at a density of 1 × 10^4^ cells/well and incubated for 24 h. Serial dilutions of MK-2048 PNP film dissolved in 1 mL of saline were added to TZM-bl cells followed by TCID_50_ of 3000 HIV-1_BaL_. After 48 h at 37 °C in 5% CO_2_, luminescence was measured using Bright-Glo (Promega, Madison, WI, USA). Luminescence of the cells treated with the film dilutions was compared to virus controls which were only treated with the cell culture media. Free MK-2048 API was dissolved in dimethyl sulfoxide (DMSO) and then serial diluted with media. The MK-2048 API group was also tested in this assay as the free drug control group.

### 2.9. PNP Permeability Study Using Transporter-Overexpressed Cell Lines

MDCKII transfected cell lines (MDR1, passages 25–30; BCRP, passages 72–80) were used in the bidirectional transport study. Cells were seeded on collagen-coated microporous polycarbonate membrane filters (0.4-µm pore size, 12-mm diameter; 12 wellTranswell plates; Costar, Corning, Corning, NY, USA). The seeding density was 5 × 10^5^ cells/insert. Cells were incubated at 37 °C in 5% CO_2_ for 3–5 days with replacement of full culture media every other day. The transepithelial electrical resistance (TEER) was measured using a Millicell ERS volt ohmmeter (Millipore, Burlington, MA, USA) every other day and before and after the transport experiments. Right before the experiment, cells were washed with prewarmed phosphate-buffered saline (PBS). Cells on the donor side were incubated with MK-2048 API (dissolved in DMSO) or MK-2048 PNP in Hank’s balanced salt solution (HBSS) while only HBSS was added to the receptor side. For the apical-to-basal (A-to-B) transport assay, the donor sides are the apical compartments, and the receptor sides are the basal compartments. It was reversed for the basal-to-apical (B-to-A) transport assay. Aliquots of 200 µL HBSS were collected from the receptor sides at 0, 15, 30, 45, 60, 75, 90, and 120 min. Drug content was assessed for permeability using LC-MS/MS (described in Section 2.4). The apparent permeability coefficient (*P_app_*) in cm/s was calculated in Equation (2), where *dQ/dt* is the rate of drug permeation in ng/s, A is the surface area of the cell layers in cm^2^, and *C*_0_ is the initial drug concentration on the donor sides in ng/mL.

(2) $$P_{app}=\frac{\frac{dQ}{dt}}{A\times C_{0}}$$

MK-2048 efflux ratio was calculated using Equation (3). *P_app_* (B − A) is the apparent permeability coefficient from the basal-to-apical direction and *P_app_* (A − B) is the apparent permeability coefficient from the apical-to-basal direction.

(3) $$\mathrm{ER}=\frac{P_{app}\left(B-A\right)}{P_{app}\left(A-B\right)}$$

### 2.10. PNP Film Permeability Study Using the Ex-Vivo Ectocervical Tissue Model in an In-Line Set-Up

Human excised ectocervical tissues were obtained from the University of Pittsburgh Biospecimen Core under IRB Protocol PRO09110431. Tissue samples were from healthy volunteers undergoing routine hysterectomy for non-cervical issues. Fresh cervical tissues were snap-frozen and stored at −80 °C for future use. Before performing the ex vivo tissue permeability study, tissues were defrosted within bags in a 37 °C water bath and then transferred to cold DMEM. A section of each tissue was retained for histological evaluation. Excess stromal tissue was removed, and the epithelial layer was isolated using a Thomas-Stadie Riggs tissue slicer by a longitudinal slice through the entire tissue. Tissue thickness was measured by placing the tissue between two pre-measured slides using a digital caliper. Tissue permeability studies were conducted in the In-line Cell system (Permegear, Inc., Hellertown, PA, USA). Isolated epithelial sections of the tissue were placed between the donor and receptor compartments of the In-line Cell with the epithelial side of the tissue oriented toward the donor compartment and 0.28 cm^2^ surface area of tissue exposed to the drug. The tissue-loaded In-line cells were maintained at 37 °C throughout the experiment. Free MK-2048, MK-2048 PNP lyophilized powder, and 6 mm diameter punch MK-2048 PNP film were suspended in 450 µL of vaginal fluid simulant (VFS) and placed in the donor compartment (protected from light). Donor samples were collected at the beginning and the end of the study. DMEM was used as the receptor medium with a flow rate of 50 μL/min. Receptor samples were collected at time points of 1, 2, 3, 4, 5, and 6 h. Donor and receptor samples were tested using LC-MS/MS, for MK-2048 content. The pre-and post-exposure tissues were evaluated for changes in epithelium morphology using Hematoxylin and Eosin (H&E) staining as described before [34].

### 2.11. In Vivo Pharmacokinetic (PK) Evaluation of PNP Films in a Macaque Model

A nonhuman primate (NHP) model was utilized to evaluate the drug delivery of PNP films in in vivo cervicovaginal environments. 

Prior approval for these experiments was obtained from the University of Washington Institutional Animal Care and Use Committee (IACUC) and the Washington National Primate Research Center (WaNPRC), where the animals were housed in animal biosafety level (ABSL)-2 facilities (general colony housing). The animals were housed and cared for under conditions that meet the National Institute of Health (NIH) standards as stated in the Guide for the Care and Use of Laboratory Animals [35], the Institute for Laboratory Animal Research (ILAR) recommendations, and the American Association for Accreditation of Laboratory Animal Care (AAALAC) accreditation standards for animals of this species. The University of Washington, including the Washington National Primate Research Center, is fully accredited by AAALAC.

The tested film products (1” × 1”) were administered to four macaques (*Macaca nemestrina*) on the same day. Film retention was assessed as it dissolved in the macaque vagina. Colposcopy-aided photography was used to document the appearance of the film product as long as it was distinct from vaginal exudate. Tissue samples were collected before and after the insertions. Blood for plasma, vaginal fluids, and tissues were sampled daily for two weeks, then twice weekly for the next four weeks, to determine MK-2048 presence as a measure of drug delivery. Vaginal pH was also monitored throughout the study. 

All samples collected for PK analyses were frozen (−80 °C) until analyses [19]. In short, vaginal fluid swab samples were thawed to ambient temperature and visually inspected. 1 mL of blank K2 EDTA human plasma was added to the cryovial tube and vortexed for 10 s. Samples were then placed onto a shaker oscillating at 600–900 times/minute for 90 min in a cell incubator (37 °C) prior to overnight incubation. After incubation, swabs were removed, and the remaining sample was equilibrated to ambient temperature before plasma assay extraction. For tissue samples, individual tissue was homogenized in 1 mL of collagenase/Li-Heparin plasma solution. After homogenization, samples were diluted 2× with K2 EDTA human plasma. Further dilutions with plasma would be performed as needed before plasma assay extractions. A liquid-liquid extraction (LLE) assay was performed on all processed samples including the blood plasma samples. Briefly, 200 µL of the sample was mixed with 400 µL of 0.5 M NH_4_OAc in an individual 12 × 75 glass tube, before adding 2 mL of methyl tert-butyl ether (MTBE). Samples were vortexed for 10 s and then centrifuged at 2000× *g* for 5 min. After centrifugation, tubes were carefully placed into an IPA-dry ice bath without disturbing the aqueous/MTBE layer. Once the aqueous layer was frozen, upper layer samples were collected and dried in a Turbovap dryer at 40 °C. Samples were reconstituted with 100 µL of 40% methanol in water, vortex mixed, and analyzed with LC-MS/MS.

### 2.12. Statistical Analysis

All values are reported as means ± standard deviation (SD). Statistical data analyses were performed using the student’s *t*-test between two groups and one-way ANOVA with Tukey’s post hoc test for the analysis of three or more study groups, with *p* < 0.05 as the minimal level of significance, *p* < 0.01 for very significant and *p* < 0.001 for highly significant. All tests were performed using the GraphPad Prism software version 9.

## 3. Results

### 3.1. Characterization of MK-2048-Loaded PLGA Nanoparticles (PNPs) 

**Figure 1:**
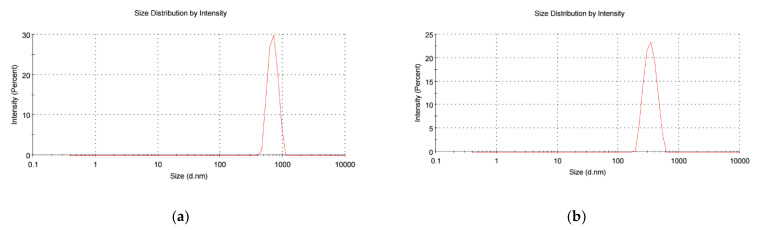
Size distribution tested by DLS measurements for PNP samples (**a**) before lyophilization and (**b**) after lyophilization. Samples were tested in triplicate. One representative result is shown for each group. The x-axes are presented on a logarithmic scale.

The PNP suspension was characterized for hydrodynamic diameter as an in-process quality control measure. The mean diameter was found to be 549 ± 51 nm (N = 3) before lyophilization. (Figure 1a).

After lyophilization, the dry PNP powder was resuspended in water. Samples were found to have a hydrodynamic diameter of 382 ± 19 nm (N = 3), with a zeta-potential of -15.2 mV. The poly-dispersion index (PDI) of PNP was 0.26 ± 0.04. A representative size distribution figure shows the size distribution of PNPs after lyophilization (Figure 1b). Lyophilized PNPs have desired size for vaginal delivery (200–500 nm [36]), negative charge, and relatively low polydispersity. 

**Figure 2:**
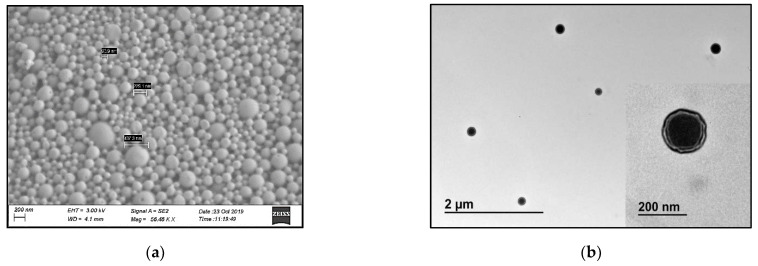
(**a**) SEM and (**b**) TEM images of the MK-2048-loaded PNPs. Scale bars are shown on each image. The TEM image consists of two scales with a close-up of one PNP inserted.

The spherical shape of PNP was confirmed by SEM and TEM, as shown in Figure 2. TEM image (shown in Figure 2b insert) supports a corona-core nanoparticle structure, where MK-2048 is expected to be loaded within the core matrix, while PVA coats the particle surface.

The final PNP achieved a very high encapsulation efficacy of 95%. In addition, the tested drug loading (DL%) (16.40% ± 0.24%) is very close to the theoretical DL% (16.02%), indicating that MK-2048 was well incorporated into the PLGA nanoparticles.

### 3.2. Characterization of PNP-Incorporated Polymeric Films

Toward the development of a dosage form appropriate for vaginal administration, the PNPs were incorporated into a PVA-based film platform. Physical characterizations, including weight, thickness, appearance, puncture strength, and water content were evaluated for the PNP films. The PNP-incorporated film was 1” × 1” in size, translucent, flexible, and odorless. Films were found to have an average weight of 138.45 (±8.92) mg and thickness of 0.37 (±0.03) mm. On average, the puncture strength of the films was 1.82 (±0.66) kg/mm, and the water content was 4.07% (±0.30%). All the physical parameters were comparable with other vaginal polymeric films from our laboratory [37]. The drug loading (DL%) is the weight/weight ratio of MK-2048 to the film weights, which was 6.94% ± 0.12% tested by HPLC. The content uniformity evaluated by assaying 6 individual units of PNP films from different sheets and locations showed a relative standard deviation (RSD%) of 1.70%, meeting the criteria (less than 10%).

In addition, to determine the compatibility of the PNP films with vaginal microbiota of interest, PNP films were also tested in a standard microbicide safety test with two strains of *L. crispatus* and two strains of *L. jensenii* which are species common in an optimal *Lactobacillus* dominant vaginal microbiome. The log value differences of all four bacteria counts are summarized in Table 1, showing less than 1.0 in log differences between groups treated with PNP film and buffer-only control groups. The results detected no viability loss for all four *Lactobacillus* strains with MK-2048 PNP films, indicating compatibility with *Lactobacillus* species.

### 3.3. PNP Film Efficacy against HIV-1

**Figure 3:**
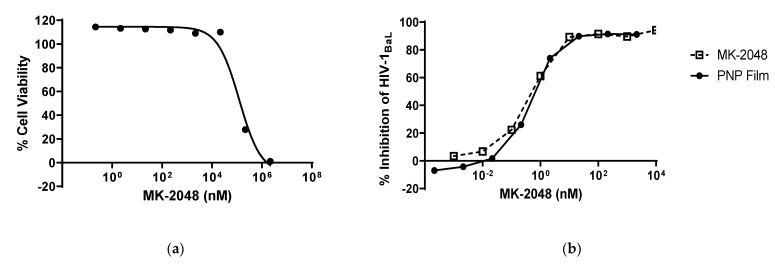
PNP film efficacy study using the TZM-bl antiviral assay. (**a**) CT_50_ of the MK-2048 PNP Film Cell Viability Assay. (**b**) MK-2048 PNP Film HIV-1 Inhibition Assay IC_50_. CT_50_: concentration required to kill 50% of the exposed cells; IC_50_: concentration required to achieve 50% inhibition of HIV.

A TZM-bl antiviral assay was used to assess the efficacy of PNP films. First, the cytotoxicity of PNP films was tested in the cell line. The concentration required to kill 50% of the exposed cells is defined as CT_50_. As shown in Figure 3a, the PNP film solution did not have any cytotoxicity up to 10 µM of MK-2048, and the CT_50_ of the PNP film was calculated to be 124.45 µM.

Subsequently, the HIV-1 inhibition assay was performed at a starting concentration that was not cytotoxic (>70% cell viability). Free MK-2048 was used as a control, and the inhibition studies were performed for the MK-2048 PNP film (Figure 3b). The concentration required to achieve 50% inhibition of HIV was defined as IC_50_. All three tests on MK-2048 PNP films resulted in similar efficacy profiles, with a mean IC_50_ of 0.46 nM. Compared to the MK-2048 free API group, there is no statistical difference in IC_50_ (0.46 nM vs. 0.54 nM), indicating that the nanoparticle-in-film technology does not impact the efficacy of MK-2048.

### 3.4. MK-2048 in PNPs Evades Transporters

**Figure 4:**
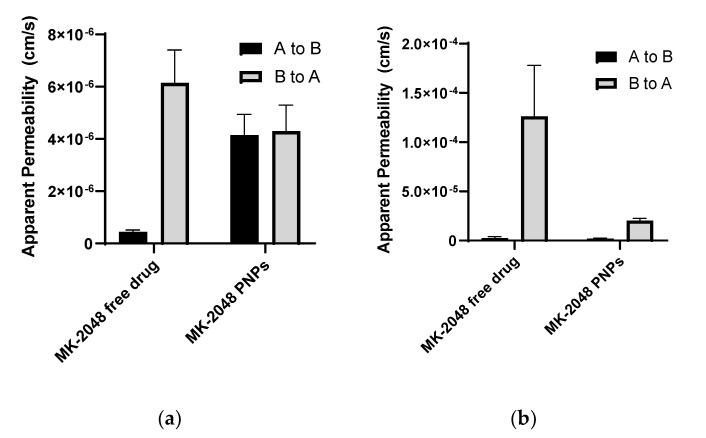
Apparent permeability (P_app_) in the transporter overexpressed cell lines. (**a**) MDCKII BCRP cell line (N = 3); (**b**) MDCKII MDR1(P-gp) cell line (N = 6). Results are reported as Mean ± SD.

Since MK-2048 was reported to be a substrate of efflux transporters [19,20], two transporter-overexpressing cell lines were utilized for permeability studies to investigate whether the nanoparticles can prevent MK-2048 from being effluxed. P_app_ values were measured for both A-to-B and B-to-A directions, and calculated efflux ratios are summarized in Table 2. For the BCRP-overexpressing cell model (MDCKII BCRP), the efflux ratio (ER) for free MK-2048 is 13.77, and for PNP 1.04. This model validates that MK-2048 is a substrate for BCRP. In addition, by incorporating MK-2048 in nanoparticles, the apparent permeability of A-to-B was increased by 10 times, resulting in increased drug content in the receptor sides (Figure 4a). For the P-gp overexpressing cell model (MDCKII MDR1), ER for free MK-2048 is 48.38, and for PNP 10.48. Even though the apparent permeability of A-to-B in both groups is similar, the Papp of B-to-A in the PNP group decreased by 10 times (Figure 4b). This means that less MK-2048 was transported into the apical side in the PNP group. In both models, the ER for free MK-2048 is higher than the PNP (13 times higher in BCRP and 4 times higher in P-gp), showing that PNP can prevent the efflux of MK-2048.

### 3.5. Ex Vivo Permeability in Ectocervical Tissues

**Figure 5:**
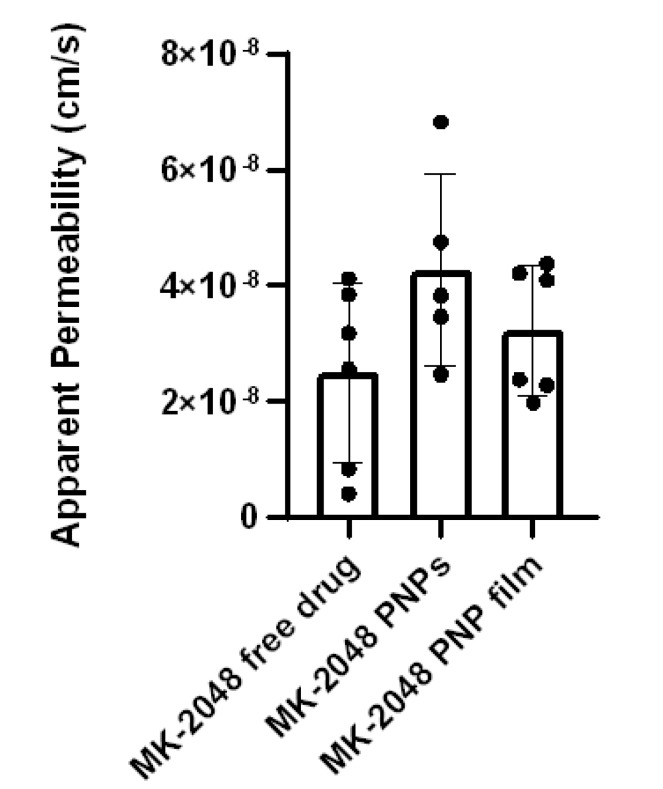
Ex vivo tissue permeability using In-Line Cell system for free MK-2048 (N = 6), MK-2048 PNP (N = 5), and MK-2048 PNP film (N = 6). Results are reported as Mean ± SD.

PNP films were evaluated in a more biologically relevant ex vivo model using human ectocervical tissues. Figure 5 shows the P_app_ of all three groups. Although not statistically significant, MK-2048 PNP and PNP Film groups both achieved higher P_app_ values compared to the free MK-2048 group, showing better tissue penetration potentials.

**Figure 6:**
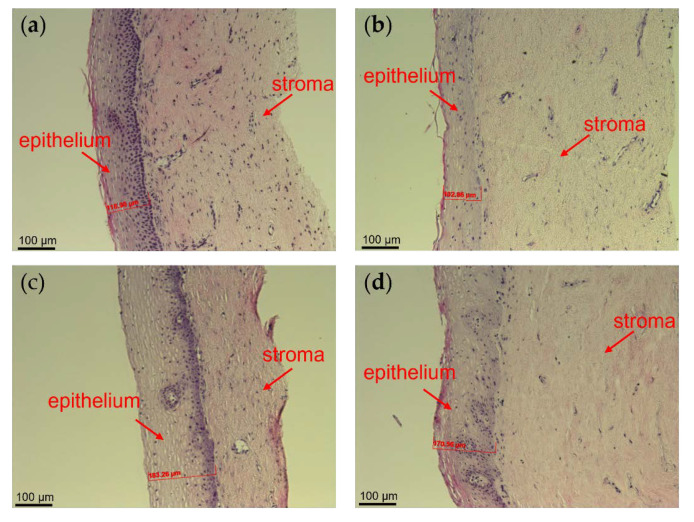
H&E staining for the morphology of human cervical tissues: (**a**) pre-treatment, (**b**) post-treatment with free MK-2048, (**c**) post-treatment with MK-2048 PNP, and (**d**) post-treatment with MK-2048 PNP film. Epithelium thickness was measured as shown in respective images. Nuclei were stained in blue.

To study if PNP films impact the epithelium integrity, cervical tissues were harvested before and after the 6-h treatments. Tissue morphologies were evaluated using H&E staining. Representative images are shown in Figure 6. There was no damage observed in the epithelium after 6-h treatments for all the groups, suggesting that no gross morphological damage will occur with vaginal use of MK-2048 PNP and PNP films.

### 3.6. MK-2048 Delivered by PNP Films Sustained in the Macaque Vagina

**Figure 7:**
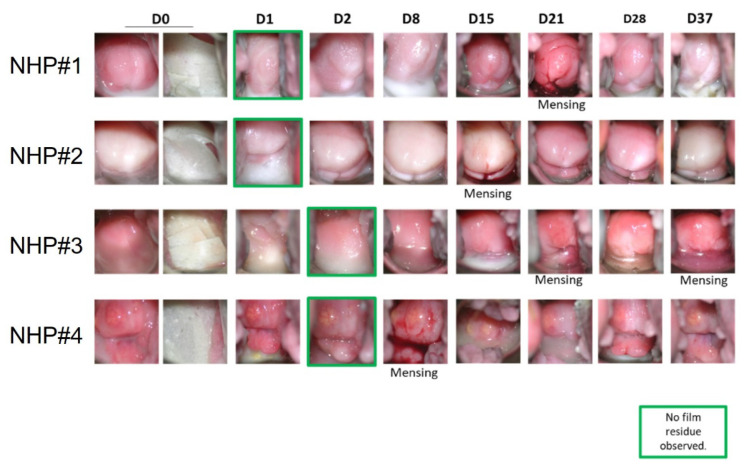
Visual observation of the MK-2048 PNP film in non-human primates (NHP) by colposcopy. Films inserted on Day 0. Green boxes indicate the day when no film residue can be observed. Menses were recorded and shown in the figure. Four non-human primates (NHP) were numbered as #1, #2, #3, and #4.

We assessed the in vivo drug release and pharmacokinetics (PK) profile of the PNP films using the macaque model. Colposcopy showed no indication of toxicity and observed film residues for up to 2 days (Figure 7). The vaginal pH was also monitored in the study since normal pH indicates healthy vaginal conditions. The normal pH in macaques ranges from 5.0–8.0. The measured pH from all four animals fluctuated within this normal range, with the majority pH ranging from 6.0 to 7.5. No significant adverse effects were observed throughout the entire study, supporting the safety profile of the PNP films. 

**Figure 8:**
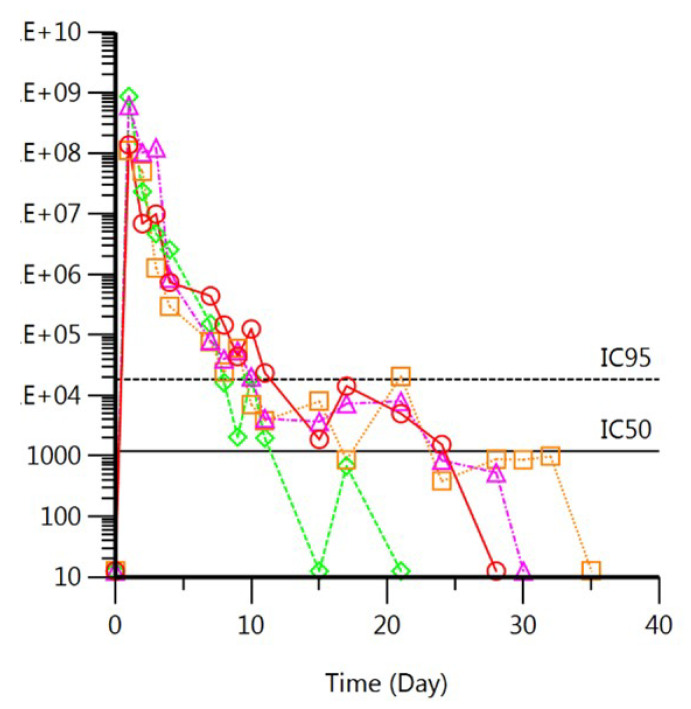
In vivo drug delivery profiles of MK-2048 PNP films (containing 10 mg MK-2048) in vaginal fluids. For numbers in the y-axis larger than 1000, E + n means 1 × 10^n^. Films inserted on Day 0. The solid line indicates the IC_50_ of MK-2048. The dash line indicates the IC_95_ of MK-2048 [38]. 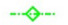: NHP#1, 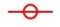: NHP#2, 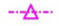: NHP#3, 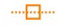: NHP#4.

Films were absent on Day 1 in two of four macaques and absent on Day 2 in the other two macaques (Figure 7), confirming the quick-dissolving nature of the film. More importantly, the MK-2048 PNP was completely dispersed in the vaginal fluids within days of application. As shown in Figure 8, the MK-2048 concentration detected in the vaginal fluids was higher than the 95% inhibitory concentration (IC_95_) level (IC_95_ = 40 nM [38]) for at least 8 days in all four macaques. The concentration remained above the 50% inhibitory concentration (IC_50_) level (IC_50_ = 2.6 nM [19]) for up to 3 weeks (in 3 of the 4 macaques). Data suggested that MK-2048 circulations were prolonged in the vaginal fluids even after the base film dissolved.

## 4. Discussion

Integrating nanomedicine and film technology renders a dosage form that is discrete, non-leaky, and portable, while allowing the flexibility of nanosystems, i.e., high drug loading, tunable drug release, and improved mucosal delivery. Nanoparticle-in-film technology has been investigated previously to achieve higher tissue drug concentrations due to the nanoparticle’s tunable size and surface properties that can achieve enhanced release, mucoadhesion, and penetration [28,29,39,40,41]. Sarmento et al. and das Neves et al. have demonstrated that the nanoparticle-in-film technology can achieve a 24-h extended-release of tenofovir in a mouse model [28,29]. In order to reduce dosing frequency and potentially increase user adherence and acceptability [30,31], there is a need for a longer extended-release. Therefore, in this study, we engineered a long-acting film dosage form and evaluated its properties and performance in in vitro, ex vivo, and in vivo models. The film was designed to dissolve quickly and release the nanoparticles, which will provide extended-release of MK-2048 for weeks and remain discrete.

The main limitation for long-acting vaginal delivery through nanosystems is the physiological removal that occurs by vaginal fluids [36]. To overcome this barrier, recent studies have taken advantage of mucus-penetrating approaches, by manipulating the size and surface properties such as charge and polymer composition [36,40,41,42]. A study reported that the space between mucin fibers (pore size) is around 340 nm [43]. Thus, for mucus-penetrating nanoparticles (MPNPs), it is desired to have an average size of 200–500 nm [36,39]. The charge of nanoparticles is another important consideration because the mucins, which are the main functional component of cervicovaginal mucus, are negatively charged glycoproteins [36]. Positively- or negatively-charged nanoparticles may have different interactions with mucus. Carter et al. [44] observed a significantly higher transport rate for negatively charged nanoparticles compared to neutral or positively-charged ones across gastrointestinal mucus. Thus, nanoparticles with a negative surface charge may have deeper penetration in vaginal mucus. Therefore, the nanoparticles in this study were fabricated to obtain an average size of 382.23 nm, negative zeta potential (−15.2 mV), and a relatively low polydispersity (PdI: 0.26 ± 0.04), supporting their potential for increased mucus penetration. Importantly, the encapsulation efficiency was found to be high (>95%), with experimentally determined drug loading in the nanoparticle being similar to calculated theoretical values. 

The incorporation of nanoparticles into films did not alter the physicochemical properties of the films nor the demonstrated in vitro HIV inhibitory activity. In this study, the PNP-loaded films were found to be thin, translucent, soft, and flexible, which are critical physical and tactile attributes shown above to be preferred by women [17,18]. These physiochemical characterizations are also comparable to other polymeric vaginal films [37,45], including one nanoparticle film product. In addition, in vitro efficacy study (Figure 3b) showed no significant difference in the IC_50_ between free MK-2048 and the PNP films, indicating that the MK-2048 retains its pharmacological activity after releasing from nanoparticle or the film formulation.

The vaginal microbiome associated with a lower risk of HIV is dominated by *Lactobacillus* species, which produce hydrogen peroxide and maintain an acidic vaginal pH that is critical for the maintenance of homeostasis [46,47,48]. Moreover, preservation of the integrity of the vaginal mucosa could decrease the direct contact of HIV with basal epithelial cells that are more susceptible to viral transcytosis [47]. Therefore, vaginal delivery products should demonstrate good safety properties, including *Lactobacillus* compatibility, maintenance of the vaginal pH and epithelial integrity, and low cytotoxicity. MK-2048 PNP films were tested with four *Lactobacillus* strains. The reduction of *Lactobacillus* viability was less than 1.0 in log value with the presence of PNP films, demonstrating their good compatibility with the vaginal microflora of interest. Upon vaginal exposure in the in vivo macaque model, no changes from normal vaginal pH were observed. The results showed that the normal vaginal pH (mean ± SD = 7.3 ± 0.9, range = 5.5–9.5) [49] was maintained after PNP film administration and throughout the study duration. Neither MK-2048 PNP nor MK-2048 PNP film affected the integrity of ex vivo human ectocervical tissues after 6-h treatments, demonstrated by the histological evaluation of exposed tissues (Figure 6). This safety profile is also supported by the in vitro studies, showing no cytotoxicity at the proposed dosing levels (Figure 3a). Altogether, these results indicated that the PNP films, as well as PNPs, are not associated with significant changes in the cervicovaginal environment including microbiota, pH, and epithelial integrity, which are considered natural barriers against HIV infections [41,47,48].

The long-acting effect of the PNP films was hypothesized to be achieved by sustained drug release and evasion of transporters. Integrase inhibitors such as raltegravir and elvitegravir are substrates of efflux transporters existing in the vaginal tissues, namely P-gp and BCRP [22,23,24,25]. The second-generation integrase inhibitor, MK-2048, was also identified to be a substrate of efflux transporters. Therefore, in vitro bi-directional cell assays demonstrated higher ERs for free MK-2048 compared to MK-2048 PNPs, indicating that nanoparticles can potentially evade the transporters. This evading effect was also partially demonstrated in the ex vivo study where MK-2048 permeability was increased by 1.7- and 1.3- fold for the PNPs and PNP films, respectively.

A proof-of-concept in vivo study was conducted in pigtailed macaques to assess the sustained drug release of nanoparticles in the cervicovaginal environment. This NHP model has anatomical and physiological similarities to humans, which allows a more reliable translation of results to humans than in vitro studies. Based on visual observation and imaging, the films were found to quickly dissolve within 1–2 days after insertion (Figure 7). With film dissolving, PNPs should be released and dispersed in the vagina within days. The PNPs were suspected to circulate in vaginal fluids and release MK-2048. Results in Figure 8 demonstrated the MK-2048 levels in vaginal fluid above the IC_50_ for at least 3 weeks in 3 (out of 4) macaques, indicating sustained MK-2048 release from the nanoparticles. The plasma levels remained low with 2 out of 4 macaques, showing levels above IC_50_ only for 2 days. Surprisingly, despite sustained vaginal levels being found, no appreciable concentrations were found in cervical and vaginal tissues. Although more investigations are warranted, we suspect that the nanoparticles are likely to traverse the non-adherent mucus to reach the adherent mucus, where they slowly released MK-2048. Given the tight tissue junctions differences among models, the superiority of nanoparticles shown in vitro was only partially translated in vivo. Therefore, there is still further optimization needed for PNPs, such as size and surface properties [36,50], to enable penetration into the vaginal epithelium without damaging the integrity [41]. Nevertheless, this study was the first to demonstrate sustained drug release in the vagina for several weeks using nanoparticles in a large animal model.

This study also enlightened future improvements for the current nanoparticle-in-film technology. Nanoparticle surface modifications should be further explored. Firstly, polyethylene glycol (PEG) coated nanoparticles should be considered in this NPs-in-film technology. Studies showed that PEG-coated NPs can diffuse across human mucus rapidly and achieve better mucosal epithelium penetration [39,51]. Thus, PEGylation is a good strategy to improve nanoparticle-based drug delivery. Secondly, thiomers (thiolated polymers) can also be considered for surface modifications. Thiomers are reported to penetrate mucus, bind with endothelial cells, and provide sustained release [52,53]. Thus, thiomer-coated NPs can be used as mucus-adhesive nanoparticles (MHNPs). Literature [54] showed encouraging results of MHNPs in mice, where they had a longer vaginal retention time and more rapid internalization by vaginal cells. In addition, our lab has previously reported sustained drug release from a thiomer-based film platform for dapivirine and levonorgestrel [37]. Based on these studies, the nanoparticle surface could be modified with thiomers to render mucoadhesion. Lastly, a combination of MPNPs-in-mucoadhesive-base-film could be explored [36,37]. The platform will have dual functions consisting of mucus-penetrating nanoparticles released from a bio-adhesive formulation. This concept was explored by Sarmento et al. [29] and demonstrated feasibility and safety in mice. However, further optimization and assessments are needed to advance the platform. 

## 5. Conclusions

PNPs and nanoparticle-in-film technology displayed promising attributes including high drug encapsulation efficiency, lack of toxicity, and efficacy. The results from in vitro, ex vivo, and in vivo models provide preliminary evidence supporting the safe vaginal use of PNPs and PNP films. Importantly, this study was the first to demonstrate sustained drug release in the vagina for weeks using nanoparticles in a large animal model. The current nanoparticle-in-film technology serves as a promising drug delivery system for the development of vaginal anti-HIV microbicides and can serve as an extended-release drug delivery dosage form to reduce dosing frequency to overcome user adherence issues. The results support the potential of nanosystems for vaginal applications and lay the groundwork for future research to further modify and advance this platform.

## Figures and Tables

**Table 1:** *Lactobacillus* compatibility with the PNP Films.

Lactobacillus Strain	Film–Buffer Log Difference
*L. crispatus* CTV05	0.062
*L. crispatus* ATCC 33197	0.560
*L. jensenii* ATCC 25258	0.041
*L. jensenii* LBP 28Ab	−0.214

**Table 2:** Efflux Ratio (ER) values in two transporter-overexpressed cell lines.

ER	MK-2048 Free Drug	MK-2048 Nanoparticles
BCRP	13.77	1.04
P-gp	48.38	10.48

## Data Availability

Not applicable.
